# An online learning algorithm for adapting leg stiffness and stride angle for efficient quadruped robot trotting

**DOI:** 10.3389/frobt.2023.1127898

**Published:** 2023-04-06

**Authors:** Mahtab Aboufazeli, Ali Samare Filsoofi, Jason Gurney, Sanford G. Meek, V John Mathews

**Affiliations:** ^1^ School of Electrical Engineering and Computer Science, Oregon State University, Corvallis, OR, United States; ^2^ Department of Mechanical Engineering and the Robotics Center, University of Utah, Salt LakeCity, UT, United States

**Keywords:** adaptive control, bio-inspired robots, variable passive compliance, efficient legged robots, online learning algorithms

## Abstract

Animals adjust their leg stiffness and stride angle in response to changing ground conditions and gait parameters, resulting in improved stability and reduced energy consumption. This paper presents an online learning algorithm that attempts to mimic such animal behavior by maximizing energy efficiency on the fly or equivalently, minimizing the cost of transport of legged robots by adaptively changing the leg stiffness and stride angle while the robot is traversing on grounds with unknown characteristics. The algorithm employs an approximate stochastic gradient method to change the parameters in real-time, and has the following advantages: (1) the algorithm is computationally efficient and suitable for real-time operation; (2) it does not require training; (3) it is model-free, implying that precise modeling of the robot is not required for good performance; and (4) the algorithm is generally applicable and can be easily incorporated into a variety of legged robots with adaptable parameters and gaits beyond those implemented in this paper. Results of exhaustive performance assessment through numerical simulations and experiments on an under-actuated quadruped robot with compliant legs are included in the paper. The robot platform used a pneumatic piston in each leg as a variable, passive compliant element. Performance evaluation using simulations and experiments indicated that the algorithm was capable of converging to near-optimal values of the cost of transport for given operating conditions, terrain properties, and gait characteristics with no prior knowledge of the terrain and gait conditions. The simplicity of the algorithm and its demonstrably improved performance make the approach of this paper an excellent candidate for adaptively controlling tunable parameters of compliant, legged robots.

## 1 Introduction

Mobile robots are employed for a wide range of applications and in various environments. Energy efficiency of mobile robots is a critical issue as they carry their power sources on them, and batteries must comply with space and weight constraints. Unlike wheeled robots, legged robots can mimic animal behavior and adapt to unstructured environments that are inaccessible to wheeled robots.

Animals adjust their leg compliance and gait parameters such as the stride length and stride frequency of their gaits to traverse more efficiently in response to environmental changes and/or their locomotion speeds. Inspired by biology, this paper presents strategies to adaptively reduce the energy consumption of legged robots during gait by continuously adjusting leg stiffness and stride angle. Although we consider only the adaptation of leg stiffness and stride angle, the algorithm can be easily generalized to other parameters of the robot design and of the gaits.

Passive compliance in distal legs of animals improves stability and reduces energy consumption (Alexander, 1984). Animals can store energy at the beginning of the stance phase from touchdown to midstance and release it for use in the rest of the stance phase (Dickinson et al., 2000). Geyer et al. (2006) have demonstrated that a compliant leg model is needed to characterize normal walking and running mechanisms in humans. Human runners increase their leg stiffness on soft grounds and decrease it on hard grounds (Ferris and Farley, 1997; Ferris et al., 1998). Humans also increase their equivalent leg stiffness with increasing running speeds (Kim and Park, 2011) and with increasing stride frequencies (Farley and Gonzalez, 1996). The stride length and stride rate of human runners increase with increasing forward velocity (Elliott and Blanksby, 1979). Animals also increase their stride length proportionately to their forward speeds (Jayes and Alexander, 1978; Barrey et al., 2002). Experimental analyses on humans and other bipedal and quadruped animals have shown that they vary their stride lengths while changing their forward velocities based on the anatomy of their bodies and their legs (Alexander, 2004). It has been argued, based on experimental results, that changing the stride length and stiffness of the legs in various gaits and speeds conserves energy (Elliott and Blanksby, 1979; Umberger and Martin, 2007; Shen and Seipel, 2015).

Inspired by the biological phenomena described above, this paper presents an online learning algorithm to continuously adapt the leg stiffness and the stride length of a quadruped robot in order to reduce its energy consumption during locomotion. These parameters were updated using a block gradient algorithm with the goal of reducing the cost of transport (CoT) (Kottege et al., 2015). CoT is a non-dimensional metric used to compare the energy efficiency of the transportation of different mobile robots and/or animals. CoT is defined as the consumed power per 1 kg weight of the robot/animal to traverse a distance of 1 m.

The leg stiffness of the quadruped robot was changed in real time by varying the pressure in a pneumatic piston located in each distal leg segment, and the stride length was adjusted by manipulating the stride angle. Our approach has many advantages over the state-of-the-art, including: (1) it is computationally efficient and can be easily implemented in real-time; (2) it does not need to train the robot in advance; (3) it is model-free, implying that precise modeling of the robot is not required for good performance; and (4) it can be applied to a variety of legged robots and gaits beyond those we implemented.

The performance of the online learning algorithm was evaluated using a large set of simulations and experiments performed on an under-actuated quadruped platform (Gurney et al., 2023). The results presented in the paper indicate that similar to biological phenomena, our approach adapts the leg stiffness and stride angle appropriately to achieve better energy efficiency in different operating conditions.

The rest of the paper is organized as follows. Section 2 provides a review of previous work related to design and control approaches for improving the performance of robots including those associated with energy efficiency, robustness to disturbances, and the ability to track desired trajectories. Section 3 describes the design of the robot and the online learning algorithm. Performance evaluation of the robot using simulations and experiments is presented in Section 4. Finally, our concluding remarks are provided in Section 5.

## 2 Related work

A number of approaches to improve the energy efficiency, accuracy of trajectory tracking, robustness to disturbances, and environmental changes of legged robots have been reported in the literature.

One class of approaches was inspired by biological phenomena and targets mechanical design principles. One example involves designing robot legs with compliant actuators such as hydraulic actuators as in HyQ (Boaventura et al., 2012) and SCalf (Yang et al., 2019), and pneumatic pistons used by Mirzana et al. (2018). Employing compliant actuators improves robustness to unstructured environments for a wide range of dynamic locomotion (Boaventura et al., 2012), but cannot reduce energy consumption because such systems do not have a mechanism to store and release energy as passive compliance systems do. Hua et al. (2019) designed a hydraulic servo actuator with passive compliance (HPCA) for SCalf and used a compliance control algorithm based on a virtual model of the robot to improve its energy efficiency. A number of systems employing passive compliance in robot legs have been reported in the literature. Examples include Cheetah-cub (Spröwitz et al., 2013), StarlETH (Hutter et al., 2012), iSprawl (Kim et al., 2006), MIT Cheetah (Seok et al., 2013) ATRIAS (Hubicki et al., 2018), ANYmal (Hutter et al., 2016) and COMAN Kormushev et al. (2019) robots. Passive compliance conserves energy and improves the robot’s robustness to disturbances caused by external forces. Analyses of passive compliance systems have shown that two configurations of springs and actuators, i.e., series elastic actuators (SEA) and parallel elastic actuators (PEA) are more energy efficient compared to rigid actuators (RA), and SEA is more efficient than PEA (Verstraten et al., 2016; Kashiri et al., 2018). The optimum stiffness of the springs in such systems was obtained empirically in many designs (Kim et al., 2006; Spröwitz et al., 2013; Kormushev et al., 2019). However, the optimum stiffness varies with operating conditions. Passive compliance with adjustable stiffness is a solution to conserve energy and tune the stiffness when the operating conditions vary. For example, Koco et al. (2016) designed a variable passive compliance system that enabled a quadruped robot to adjust to grounds with different stiffness assuming that the stiffness of the ground is known. Christie et al. (2019) verified the effect of adjusting the leg stiffness on reducing the CoT by designing a robot with magnetorheological-fluid-centric variable stiffness legs.

The second class of approaches for designing high-performance legged robots involves designing parameters of the robot or gait by employing optimization algorithms to minimize energy consumption, trajectory tracking error, etc., improve the smoothness of the motion and robustness to disturbances, or achieve another appropriate objective. Many of these optimization algorithms were implemented offline, and the resulting parameters of the algorithm were kept frozen during locomotion. Examples of such algorithms include variations of policy gradient methods such as the policy gradient reinforcement learning implemented on Sony Aibo (Kohl and Stone, 2004), the policy gradient learning combined with interpolation between the policies used in Cassie (Xie et al., 2018) and the guided constraint policy gradient method used in ANYmal (Gangapurwala et al., 2020). Other than gradient-based algorithms, methods such as Bayesian optimization approaches as in (Lizotte et al., 2007) and (Calandra et al., 2014) and an archive-based micro genetic algorithm implemented on the hexapod robot in (Zhai et al., 2020) were employed to optimize the desired objective functions. Jin et al. (2013) applied a non-linear quadratic optimization approach to minimize the energy cost of a compliant hexapod robot by finding the optimal velocity and duty factor. They also showed *via* simulations that the values of duty factor, velocity, and stride angle vary for various weights and payloads. Unlike these approaches, our algorithm was implemented online and the parameters can be adapted in real time.

Some of the recent optimization approaches for controlling robot locomotion were designed to respond adaptively to environmental changes, unknown initial conditions, and/or damage. Examples include stiffness control of a simulated model of a passive bipedal robot using a double-deep Q network presented in (Wu et al., 2019) and intelligent trial and error algorithm for robots to adapt to damage presented in (Cully et al., 2015). Both algorithms require offline training. Arena et al. (2021) implemented an adaptive controller based on the FitzHugh-Nagumo neuron to reduce the energy consumption of a model of Mini MIT cheetah while the robot walked on uneven grounds. They found the optimal values of the parameters of the trajectory for five different values of slopes and employed a state machine to update the parameters of the trajectory with the optimal parameters of the closest slope. Even though their method was implemented online, they only considered five distinct slopes for one specific forward velocity, and the optimal parameters were not computed for other values of forward velocities and slopes.

Various adaptive optimization methods have been implemented on robot arm manipulators to either follow desired trajectories or identify the plant models online. These methods include fuzzy neural networks (He and Dong, 2017), adaptive fuzzy full state feedback control (Yu et al., 2020), and adaptive impedance control (Li et al., 2016). Zhou et al. employed recurrent neural networks (RNN) to control the compliance of redundant robot manipulators (Zhou et al., 2020a) and an RNN combined with an adaptive online identifier to learn the kinematic parameters of the manipulator (Zhou et al., 2020b). However, the number of state variables in multi-legged robots is much greater than in a robot arm manipulator. This increases the computational complexity of the algorithms and makes them challenging to implement in real-time.

The third class of approaches to improve the performance of the robots involve creating efficient locomotion using appropriate trajectory planning algorithms. For example, Kormushev et al. (2019) employed a reinforcement learning approach that evolved policy parametrization dynamically. Yu et al. (2018) generated symmetric trajectories using a curriculum learning method. Ordonez-Apraez et al. (2022) presented a maximum entropy reinforcement learning architecture to derive the optimal policy that minimized the energy of locomotion of two robots, one bipedal and one quadruped, in a simulation environment. Liu et al. (2015) used a particle swarm optimization method and Koco et al. (2014) employed a genetic multi-objective optimization algorithm and a pattern search method used in SCalf (Yang et al., 2019) to determine the robot’s foot trajectory. All these algorithms were implemented offline. In SoftLegs (Gasparri et al., 2018), a numerical optimization algorithm was employed to generate a dataset of optimized trajectories offline, and new trajectories were generated online by synthesizing the trajectories using the dataset. Xin et al. (2020) optimized the torque trajectory of ANYmal in the presence of disturbances on an uphill ramp and on grounds with low friction. Their controller combined a cartesian impedance control algorithm with quadratic programming under certain stability constraints and torque limits. The resulting algorithm was computationally efficient and implemented online for static walking but there was no report of implementing the system for dynamic gaits such as trotting. A combination of model-based control and model-free reinforcement learning framework was introduced in (Da et al., 2021) to adaptively learn contact sequences. However, this framework requires training on a simulated model of the robot and the authors only presented the results of implementing their algorithm on a real robot at low speeds.

The algorithm of this paper updates the parameters of the robot design and angular trajectory of the hip/shoulder joints with the goal of reducing the CoT for dynamic gaits. Unlike a number of previous algorithms that employed offline optimization methods and/or required a large training data set and hours of training on either the real robot or a model of the robot, our algorithm can be easily implemented online, and in real-time. A drawback of training a robot and using the trained parameters without adjustments during normal operations is that the performance of the robot will, in general, degrade over time. Performance degradation can arise from changes in the operating conditions as well as changes in the robot during operation. Our approach mitigates all these problems.

## 3 Methodology

This Section describes the robot platform and the online learning algorithm designed to reduce energy consumption online and in real-time.

### 3.1 Robot platform

**FIGURE 1:**
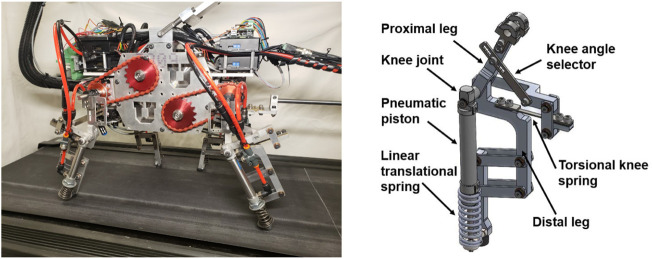
Left: A side view of UPed Gurney et al. (2023). Right: A schematic of one of the robot’s legs designed in solid works. Used with permission of ASME, from [UPed: A Quadruped Robot Platform to Study Directional Leg Compliance, Gurney and Filsoofi et al., vol 15, no 1, 2023]; permission conveyed through Copyright Clearance Center, Inc.

The online learning algorithm was implemented and tested on an under-actuated quadruped platform, UPed, designed and built at the University of Utah Gurney et al. (2023). The left panel of Figure 1 displays a picture of the robot. A schematic of one of the robot’s legs is shown separately in the right panel.

The leg design involved an elbow back and knee forward configuration, with passive compliance at the knee/elbow joints and the distal limb Lee and Meek (2005). Previous work Meek et al. (2008) has shown that this configuration reduces pitch rate compared to the elbow forward and/or knee backward designs. Lower pitch rate improves the inherent pitching stability of the robot’s gait. The robot was restricted to planar motion because each of its legs was actuated in the plane perpendicular to the main body by a DC motor located in the hip/shoulder joint. The proximal and the distal segments of the leg were connected together with a passive torsional spring. The proximal limb was a solid bar; however, the distal limb was composed of a linear translational spring in parallel with a pneumatic piston. Details of the robot design are provided in Gurney et al. (2023).

During the stance phase of each leg, a valve is closed preventing air flow in the cylinder, making it act like a passive spring. The air pressure, *P*_*td*_, in the pneumatic piston adapts and controls the stiffness of the leg. Consequently, we can use the piston as a compliant element with variable stiffness, and adjust its stiffness in real time by varying the air pressure in the piston. Using the ideal gas law, the force exerted by the parallel combination of the pneumatic piston and the linear spring during stance phase is related to their displacement as

$$F=\frac{P_{td}AL_{0}}{\left(L_{0}-x\right)}-P_{atm}A+K_{lin}x$$ (1)

where *L*_0_ and *P*_*td*_ represent the length and the pressure, respectively, of the pneumatic piston at the time of touchdown, the parameters *A*, *P*_*atm*_ and *x* denote the area of the piston, the ambient pressure and the displacement of the piston with respect to *L*_0_, respectively, and *K*_*lin*_ represents the stiffness of the linear spring. The stiffness of the passive compliance is the derivative of the force *F* with respect to the displacement *x*, and is given by

$$K=\frac{dF}{dx}=\frac{P_{td}AL_{0}}{{\left(L_{0}-x\right)}^{2}}+K_{lin}$$ (2)

The adaptive parameters of the robot are the stiffness of the legs and the stride angle of its gait. Based on the results in (1) and 2, we adapt the pressure of the pneumatic spring to control the stiffness of the passive compliance of the leg. The stride angle *r* is defined as the maximum deviation of the hip/shoulder angles from the static stance phase.

**FIGURE 2:**
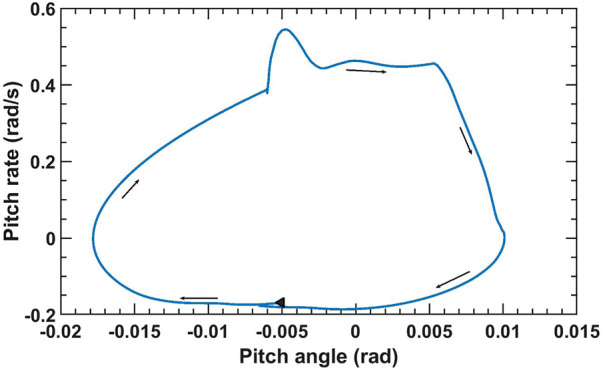
Plot of pitch rate vs. pitch angle for *r* = 14°, *P*_*td*_ = 3 × 10^5^ Pa and angular velocity *ω* = 90 deg/s. The black triangle displays the beginning of the cycle.

Our robot was restricted for planar motion in the longitudinal direction. As a result we can make inferences about the stability of the robot by analyzing its pitching motion. (Meek et al., 2008). demonstrated that the elbow-backward, knee-forward configuration results in lower pitch oscillations than three other configurations. In this work, we empirically swept through stride angle and touchdown pressure and selected a subset of all possible parameter ranges in which the robot’s pitching motion was bounded. During deployment, the adaptation of the parameters were restricted to this subset, allowing the robot to operate in a stable manner. Figure 2 displays the plot of pitch angle-pitch rate for a simulated model of the robot n MATLAB-Simulink using the Simscape Multibody library Simulink Documentation (2018) for stride angle *r* = 14° and touchdown pressure *P*_*td*_ = 3 × 10^5^ Pa which belonged to the parameter set that produced a stable gait. Since the trajectory of the pitch rate fits inside a circle with bounded radius, this periodic gait is asymptoticly stable. Furthermore, (Usherwood and Granatosky, 2020), demonstrated that the knee-forward and elbow-backward configuration reduces joint and limb work and pitch moment. This indicates a direct relationship between energy consumption and the pitch accelerations of biological systems. In addition, pitch rate and CoT had similar trends in the experiments performed on UPed (Gurney et al., 2023) for various conditions. Our algorithm adaptively reduces the energy consumption of the robot. This leads to reduced pitch acceleration and improved the stability of pitching motion of the robot.

### 3.2 Adaptation of leg stiffness and stride angle

Although easily generalized, we developed an online learning algorithm for adapting the stride angle of the robot’s gait and the stiffness of the passive compliance of its legs, with the objective of reducing the average value of the consumed energy at each time interval since our main target is to minimize the statistical expectation of a positive power of the CoT. For simplicity of implementation, the CoT is computed over an integer *N*_*b*_ number of strides. In the rest of the paper, we denote *N*_*b*_ strides as one block. Assuming that the robot has already traversed the (*m* − 1)^*th*^ block, our objective is to reduce a positive power of the CoT while the robot is traversing the *mth* block, i.e., reduce

$$E\left\{{\text{CoT}}^{d}\left(m;\underline{U}\left(m\right),\underline{\theta }\right)\right\}$$ (3)

where *E*{.} is the statistical expectation of ⋅, $\underline{\theta }={\left[r,P_{td}\right]}^{T}$ is the vector of optimization parameters consisting of the stride angle *r* and touchdown pressure *P*_*td*_ and $\underline{U}\left(m\right)={\left[\underline{U}{\left[T\left(m\right)\right]}^{T},\ldots ,\underline{U}{\left[T\left(m+1\right)-1\right]}^{T}\right]}^{T}$ represents the augmented vector of all control signals for block *m*. Let $\underline{U}\left[n\right]={\left[I_{1}\left[n\right],I_{2}\left(n\right),I_{3}\left(n\right),I_{4}\left(n\right)\right]}^{T}$ be the vector of control signals at time *n* where *I*_*j*_(*n*) is the current drawn by the *j*th motor at time n. Let block *m* start at time *T*(*m*). The CoT for block *m* is defined as Kottege et al. (2015).

$$\text{CoT}\left(m\right)=\frac{P_{avg}\left(m\right)}{MgV\left(m\right)}$$ (4)

where *P*_*avg*_(*m*) is the sum of the average power consumed by all the motors of the robot while the robot is traversing the interval of block *m*, *V*(*m*) is the robot’s average linear forward velocity in the interval, *g* is the acceleration due to the gravity, and *M* is the total mass of the robot. The average power consumed by the motor that actuates the *ith* leg for block *m* may be estimated as

$$P_{i}\left(m\right)=\frac{1}{T\left(m+1\right)-T\left(m\right)}\overset{T\left(m+1\right)-1}{\underset{n=T\left(m\right)}{\sum }}\left\{RI_{i}{\left(n\right)}^{2}+|\omega_{i}\left(n\right)R_{G}k_{t}I_{i}\left(n\right)|\right\}\Delta t\left(n\right)$$ (5)

where *R*, *k*_*t*_, and *R*_*G*_ represent the motor coil resistance, torque constant, and gearbox ratio, respectively, of the motors actuating the shoulder/hip angle movements, *ω*_*i*_ is the hip/shoulder angular velocity of the *ith* leg (after the gearbox), *I*_*i*_ is the current consumed by the *ith* motor, and Δ*t*(*n*) is the sampling period for the measurements. In (5), it is assumed that all four motors have the same values of *R*, *k*_*t*_, and *R*_*G*_.

We employed a stochastic gradient adaptation method to update the optimization parameters online and in real-time. The adaptive algorithm does not assume knowledge of the functional relationship between the parameters of the robot and the CoT. Instead, approximations of the gradients of $\text{CoT}\left(m;\underline{\theta }\right)$ with respect to the adaptive parameters are calculated numerically. The basic online learning strategy, then, is to employ update equations of the form:

$$r\left(m\right)=r\left(m-m_{r}\right)-\eta_{r}\text{CoT}\left(m-m_{r}\right)f_{r}\left(\hat{\nabla_{r}}\text{CoT}\left(m-m_{r}\right)\right)$$ (6)

and

$$P_{td}\left(m\right)=P_{td}\left(m-m_{P}\right)-\eta_{P}\text{CoT}\left(m-m_{P}\right)f_{P}\left(\hat{\nabla_{P}}\text{CoT}\left(m-m_{P}\right)\right)$$ (7)

where *f*_*r*_ and *f*_*P*_ are non-decreasing functions that are bounded above and below by finite numbers to avoid large changes of the adaptive parameters that are updated. The parameters *m*_*r*_ and *m*_*P*_ represent the number of blocks between successive updates of *r* and *P*_*td*_, respectively, and $\hat{\nabla_{r}}\mu \left(m\right)$ and $\hat{\nabla_{P}}\mu \left(m\right)$ denote the numerically calculated gradient of the mean of the CoT with respect to *r* and *P*_*td*_, respectively at the *mth* block. For every successive block, the gradient is calculated only for the parameters that are updated. Let *r* be the parameter that updates at block *m*. The gradient at block *m* is estimated as the slope of the CoT with respect to *r* as

$$\hat{\nabla_{r}}\text{CoT}\left(m\right)=\frac{\text{CoT}\left(m\right)-\text{CoT}\left(m-1\right)}{r\left(m\right)-r\left(m-1\right)}$$ (8)

Similarly, if *P*_*td*_ is updated at block *m* the gradient is estimated as

$$\hat{\nabla_{P}}\text{CoT}\left(m\right)=\frac{\text{CoT}\left(m\right)-\text{CoT}\left(m-1\right)}{P_{td}\left(m\right)-P_{td}\left(m-1\right)}$$ (9)

Although other more accurate approximations for the gradients are possible, we chose the definitions in (8) and 9 for computational simplicity. In this paper, the adaptive parameters are updated sequentially *i.e.*, only one parameter is updated during each block. Simultaneous update of the parameters causes a 100% correlation between the update directions of the parameters which constrains the changes of the parameters to be always in one direction. We avoid this problem by updating one of the two parameters during each update cycle and keeping the value of the other parameter the same as it was in the previous block. For the sequential block-wise stochastic gradient descent algorithm described above, we used hyperbolic tangent functions for *f*_*r*_ and *f*_*P*_ in (6) and 7. That is,

$$f_{r}\left(\hat{\nabla_{r}}\text{CoT}\left(m\right)\right)=\tanh\left(S_{r}\hat{\nabla_{r}}\text{CoT}\left(m\right)\right)$$ (10)

and

$$f_{P}\left(\hat{\nabla_{P_{td}}}\text{CoT}\left(m\right)\right)=\tanh\left(S_{P}\hat{\nabla_{P_{td}}}\text{CoT}\left(m\right)\right)$$ (11)

Here, *S*_*r*_ and *S*_*P*_ denote constant scaling factors of the numerically calculated gradients of *r* and *P*_*td*_, respectively, and control how quickly the incremental update values saturate. The parameters *η*_*r*_, *η*_*P*_, *S*_*r*_ and *S*_*P*_ are constant values and are chosen empirically. The update equations are tabulated in Table 1 for the case when *m*_*r*_ = *m*_*P*_ = 2.

**TABLE 1:** Block-wise stochastic gradient descent algorithm.

**Initialize:** *η*_*r*_, *η*_*P*_, *S*_*r*_ and *S*_*P*_
For block *m*: $\text{CoT}\left(m\right)=\frac{P_{avg}\left(m\right)}{MgV\left(m\right)}$
For *m* odd
$\left\{\begin{array}{c} \hat{\nabla_{r}}\mu \left(m-2\right)=\frac{\text{CoT}\left(m-2\right)-\text{CoT}\left(m-3\right)}{r\left(m-2\right)-r\left(m-3\right)}\;\\ r\left(m\right)=r\left(m-2\right)-\eta_{r}\left\{\text{CoT}\left(m-2\right)\right\}\tanh\left(S_{r}\hat{\nabla_{r}}\mu \left(m-2\right)\right)\; \end{array}\right.$
For *m* even
$\left\{\begin{array}{c} \hat{\nabla_{P}}\mu \left(m-2\right)=\frac{\text{CoT}\left(m-2\right)-\text{CoT}\left(m-3\right)}{p_{td}\left(m-2\right)-p_{td}\left(m-3\right)}\;\\ P_{td}\left(n\right)=P_{td}\left(m-2\right)-\eta_{P}\left\{\text{CoT}\left(m-2\right)\right\}\tanh\left(S_{P}\hat{\nabla_{P}}\mu \left(m-2\right)\right)\; \end{array}\right.$

For the experiments and simulations in this paper, we chose *d* = 2 in (3). For this case, we can interpret the update strategy to be using a time-varying learning rate given by *η*_*r*(*P*)_CoT (*n* − 2). Therefore, the equivalent learning rate compared to the vanilla stochastic gradient descent algorithm is *η*_*r*(*P*)_CoT (*n* − 2).

## 4 Results

A large number of simulations and experiments were performed to assess the capabilities of the online learning algorithm. Representative results presented here will demonstrate that the algorithm is capable of adapting the parameters of the robot and tracking time-varying operating environments online and achieving near-optimal performance.

### 4.1 Simulations

#### 4.1.1 Simulation setup

A model of the quadruped robot described in Section 3.1 was built in MATLAB-Simulink using the Simscape Multibody library Simulink Documentation (2018). The foot-ground contacts were modeled as point contacts. The ground at contact points was modeled as a parallel spring-damper system in each dimension Silva et al. (2013). Uneven grounds were built by perturbing the stiffness, damping coefficients, and the longitudinal slope of the ground by adding a zero-mean pseudo-Gaussian noise sequence to the nominal values of these parameters. The additive noise sequence was modeled as the output of a single-pole lowpass filter, computed recursively as

$$x_{\eta }\left(n+1\right)=\alpha x_{\eta }\left(n\right)+\sqrt{\left(1-\alpha^{2}\right)}\zeta \left(n\right)$$ (12)

Here, *ζ*(*n*) and the initial value *x*_*η*_(1) were chosen as independent and identically distributed (i.i.d) Gaussian variables with zero mean value and standard deviation equal to the standard deviation of the ground parameter. The parameter *α* in (12) belonged to the interval [0,1] and defined the spectrum of the added noise. Values of *α* closer to one resulted in smoother perturbations than otherwise.

For all the simulations described here, *α* was chosen to be 0.95 for all noise sequences. The standard deviation of the noise added to the ground stiffness and damping coefficients were 10% of their nominal values. The standard deviation of the longitudinal slope regardless of its mean value was equal to 0.5° and the transverse slope was equal to zero. A zero-mean i.i.d Gaussian noise sequence was added to the measured CoT with a standard deviation of 0.005 regardless of the value of the CoT which produced, for the simulation conditions, a signal-to-noise ratio in the range of 32 dB–38 dB.

The algorithm was implemented for various hip/shoulder angular velocities and nominal values of the ground parameters, tabulated in Table 2. In all simulation setups summarized in Table 2, the nominal value of the ground damping coefficient in all directions was chosen to be 100 N/m. The online learning algorithm updated optimization parameters *r* and *P*_*td*_ in the beginning of each block which is equal to *N*_*b*_ = 5 strides. Unless otherwise stated, all the simulations involved the robot trotting for 300 blocks. The actual duration of each simulation varied because the time of travel for each block depended on the hip/shoulder angular velocity *ω* and the stride angle *r*, and the stride angle was adapted in these simulations. The results presented are averages computed over 50 independent simulations, run for the same operating conditions. The initial choices of *r* and *P*_*td*_ were 12° and 4 × 10^5^ Pa, respectively, in all simulations involving online parameter learning. These choices resulted in the stable operation of the robot in all the simulation conditions explored here.

**TABLE 2:** Simulation setup, optimum parameter values, and steady-state statistics of the online learning algorithm.

Simulation parameters			Optimum values			Steady state statistics			
Slope	*ω*	*K*_*gnd*_	CoT	*r*	*P*_*td*_	CoT	*r*	*P*_*td*_	$\frac{\text{SS}\left(\text{CoT}\right)-\text{opt}\left(\text{CoT}\right)}{\text{opt}\left(\text{CoT}\right)}\times 100\%$
(deg)	(deg/s)	(KN/m)		(deg)	(Pa)		(deg)	(Pa)	
+6	140	100	0.3344	17.25	2.75 × 10^5^	0.3477 ± 0.0111	16.43 ± 0.60	3.23 ± 0.30	4.9
0	140	100	0.2932	18	2.75 × 10^5^	0.3080 ± 0.0104	17.88 ± 0.54	3.15 ± 0.24	5.0
−6	140	100	0.2718	18.75	3.00 × 10^5^	0.2944 ± 0.0104	18.15 ± 0.53	3.25 ± 0.27	8.3
+6	105	100	0.2705	14.75	2.5 × 10^5^	0.2748 ± 0.0089	14.85 ± 0.49	2.51 ± 0.29	1.5
0	105	100	0.2513	15.0	2.5 × 10^5^	0.2580 ± 0.0098	14.72 ± 0.46	2.71 ± 0.36	2.7
−6	105	100	0.2460	15.25	3.25 × 10^5^	0.2493 ± 0.0116	14.62 ± 0.38	3.06 ± 0.35	1.3
0	70	100	0.2098	13.5	2.00 × 10^5^	0.2249 ± 0.0089	13.03 ± 0.50	2.54 ± 0.33	7.2
0	105	20	0.2523	14.75	3.5 × 10^5^	0.2647 ± 0.0168	14.27 ± 0.48	3.42 ± 0.33	4.9
0	140	20	0.2811	18.25	3.5 × 10^5^	0.2957 ± 0.0183	17.94 ± 0.49	3.57 ± 0.28	5.2
0	70	20	0.2314	16	2.75 × 10^5^	0.2515 ± 0.0114	13.73 ± 0.43	3.75 ± 0.17	8.7

#### 4.1.2 Performance benchmarks

To establish the performance benchmarks, we determined the optimal values of *r* and *P*_*td*_ that minimized the CoT for all the conditions simulated. For each operating condition considered, we simulated the robot for 3 minutes for each value of the stride angle between 10° and 25° in steps of 0.25^◦^, and touchdown pressure between 2 × 10^5^ and 10 × 10^5^ Pa in steps of 0.25 × 10^5^ Pa. For each set of parameters, the average of the CoT values was calculated over the 3-min run. The optimal values of CoT and the two parameters are tabulated in Table 2. Similar ranges of stride angle and touchdown pressure were used in simulations involving online learning. Lower touchdown pressures increased the pitching motion of the robot excessively and resulted in unstable or close to unstable behavior. The reason is that at lower touchdown pressures, or equivalently, smaller values of leg stiffness, the force exerted by the legs may not be sufficient to provide the demanded momentum for the robot’s motion. Therefore, the minimum allowable touchdown pressure increases by increasing the forward velocity. For an angular velocity of 140 deg/s, pressure values below 2.5 × 10^5^ Pa were not simulated for the same reason. *P*_*td*_ greater than 10 × 10^5^ Pa was not feasible in the robot platform, and therefore not simulated.

**FIGURE 3:**
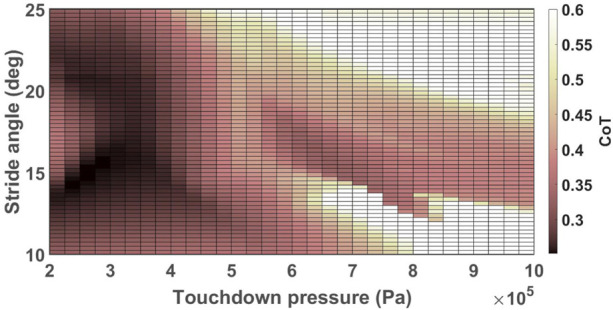
Heat map of CoT values for the robot trotting with *ω* = 105 deg/s on the ground with the stiffness of 100 ± 10 kN/m and the slope of 0 ± 0.5°. CoT values greater than 0.6 are displayed in white color to provide sufficient contrast for values closer to the optimal values.

A heat map of CoT values associated with the robot trotting on the ground with the slope of 0° ± 0.5° and stiffness of 100 ± 10 kN/m with *ω* = 105 deg/s is displayed as a function of touchdown pressure and stride angle in Figure 3. There were three local minima for this specific simulation setup corresponding to CoT values of 0.2513 when (*r*, *P*_*td*_) = (15, 2.5 × 10^5^), 0.2593 when (*r*, *P*_*td*_) = (20.75, 2.5 × 10^5^) and 0.3269 when (*r*, *P*_*td*_) = (16.5, 6.25 × 10^5^), where the units of *r* and *P*_*td*_ are degree and Pascal, respectively. The online learning algorithm can converge to any of these local minima. Exhaustive analysis of simulation results has suggested that initialization as done in our simulations may result in convergence to parameter values close to the global optimum. However, additional work is still needed to guarantee convergence to the global minima of the performance surface.

**FIGURE 4:**
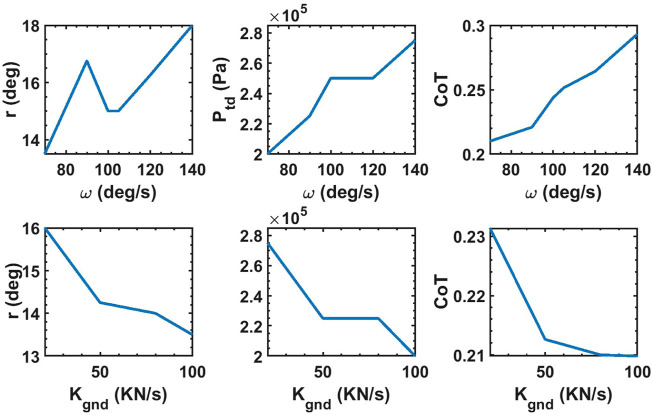
Top three plots display the optimum values of stride angle, touchdown pressure and CoT when the robot trots on uneven grounds with *K*_*gnd*_ = 100 kN/m and mean slope of zero with various hip/shoulder angular velocity. Bottom three plots display the optimum values of stride angle, touchdown pressure and CoT when the robot trots with *ω* = 70 deg/s on uneven grounds with various ground stiffness and mean slope of zero.

We can observe in Table 2 and Figure 4 that the optimal values of the CoT and the optimization parameters vary with the angular velocities of the hip/shoulder joint, ground stiffness, and longitudinal slope. Variations in the optimal values of the stride angle and leg stiffness have been reported for humans and animals for different ground conditions and forward speeds (Jayes and Alexander, 1978; Elliott and Blanksby, 1979; Farley and Gonzalez, 1996; Barrey et al., 2002; Umberger and Martin, 2007; Kim and Park, 2011; Spröwitz et al., 2013; Lussiana et al., 2015; Shen and Seipel, 2015). The trends in how changing the operating conditions such as forward velocity and ground stiffness vary the stride angle and the leg stiffness of biological models, generally comply with our observations for the simulated robot. For example, the optimum pressure is higher for faster trotting on flat ground because stronger force is needed to evoke higher velocities (Spröwitz et al., 2013). Similarly, a direct correlation between the forward speed of human runners and their leg stiffness was reported by Kim and Park (2011). The optimum stride angle of the simulated robot is higher for higher velocities similar to the results reported in (Jayes and Alexander, 1978; Elliott and Blanksby, 1979; Barrey et al., 2002) for humans and animals. The optimum touchdown pressure of the simulated robot’s leg increases on softer grounds in agreement with the biophysical data reported in (Ferris and Farley, 1997; Ferris et al., 1998). The authors of (Ferris et al., 1998) have argued that the equivalent stiffness of the leg and the ground should be constant in order to keep the same gait dynamics on various grounds. Similarly, the optimal values of touchdown pressure of the simulated robot were higher for softer grounds. There are conflicting reports of how the stride length and leg stiffness change in animals when the slope of the ground changes (Telhan et al., 2010; Lussiana et al., 2015; García-Pinillos et al., 2019). As a result, it is not possible to make a meaningful comparison of how the leg parameters of our robot and animals change on sloped terrains.

#### 4.1.3 Online learning simulations with various operating conditions

The algorithm was simulated for different choices of ground conditions and trotting speeds as tabulated in the first three columns of Table 2. The hip/shoulder angular velocities of *ω* = 140 deg/s, *ω* = 105 deg/s and *ω* = 70 deg/s, respectively, corresponded to average forward velocities of 0.65 ± 0.01 m/s, 0.44 ± 0.06 m/s and 0.30 ± 0.04 m/s, respectively. The variation in forward velocities in a condition with constant angular velocity is caused by varying the stride angle and touchdown pressure. The positive and negative signs of the ground slopes represent uphill and downhill slopes, respectively. The nominal values of the ground stiffness were chosen to be 100 kN/m (tiled floor Bosworth et al. (2016)) or 20 kN/m (mulch layer Bosworth et al. (2016)), respectively.

Table 2 also summarizes the steady-state statistics (mean ± STD) of the CoT and the adaptive parameters in these simulations. The calculations assumed that the last 100 blocks represented the steady-state and the mean results reported are the averages over the last 100 blocks and 50 independent runs. The steady-state variance of each run was calculated separately over the last 100 blocks and the square-root of the mean value of the variances are reported as the standard deviation for each case. The values of hyper-parameters i.e., *η*_*r*_, *η*_*P*_, *S*_*r*_ and *S*_*P*_ were chosen empirically by searching over a wide range of parameters. The values of *η*_*r*_ and *η*_*P*_ were empirically selected to be $\frac{0.6}{CoT\left(1\right)}$ degree for the *ω* = 140 deg/s and $\frac{0.3}{CoT\left(1\right)}\times 10^{5}$ Pa, respectively and to be $\frac{0.3}{CoT\left(1\right)}$ deg and $\frac{0.15}{CoT\left(1\right)}\times 10^{5}$ Pa, respectively, for *ω* = 105 deg/s and *ω* = 70 deg/s regardless of ground parameters. *S*_*r*_ and *S*_*P*_ were chosen to be equal to 25 regardless of the values of the angular velocity and the ground parameters. The last column of Table 2 shows the ratio of the difference between the steady-state and the optimal values of the CoT to the optimal values of the CoT for each case. The maximum deviation from the optimal value of the CoT in any of these simulations was 8.7%.

**FIGURE 5:**
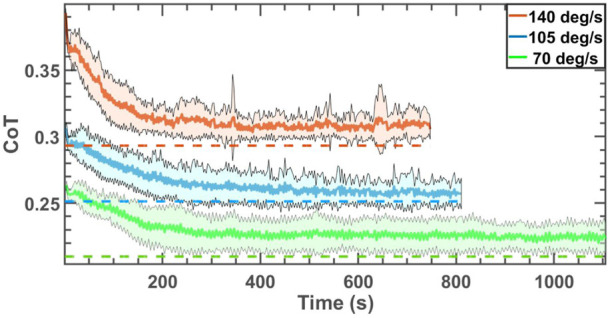
CoT of the robot trotting on uneven grounds with the mean slope of zero and three different angular velocities. The dotted lines display the optimal values corresponding to the curve with the same color. The duration of simulations is different for the three cases because the angular velocities are different for the three different cases, and the stride angle of the robot evolves differently for each operating condition.

The evolution of the average CoT computed by averaging over 50 independent simulations during each block for the simulations on uneven ground with a mean slope of 0, nominal ground stiffness of 100 kN/m, and all three angular velocities evaluated are displayed with solid lines in Figure 5. The dotted lines display the empirical optimum values of the CoT of the plots with the same colors. The shaded area surrounding each curve corresponds to the standard deviation of the CoT at each point.

Similar to the results of the experiments on human subjects running on flat, uphill and downhill slopes reported in Kim and Park (2011); Farley and Gonzalez (1996); Elliott and Blanksby (1979); Barrey et al. (2002); Jayes and Alexander (1978); Shen and Seipel (2015); Umberger and Martin (2007); Lussiana et al. (2015); Spröwitz et al. (2013), our online learning algorithm converged to different values of stride angle and touchdown pressure on different longitudinal slopes and velocities. Similar to biological models that adapt their leg stiffness on grounds with different surface stiffness Ferris et al. (1998); Ferris and Farley (1997), our algorithm converged to different values of touchdown pressure for different ground types. Although some of the minima of the performance surface may not match the observations on human and animal behavior in prior research, when the robot parameters and the CoT values converged to the global minimum, the resulting evolution of the leg parameters was similar to how animals and humans change leg stiffness and stride angle during gait.

#### 4.1.4 Robot behavior during abrupt changes in forward speed

**FIGURE 6:**
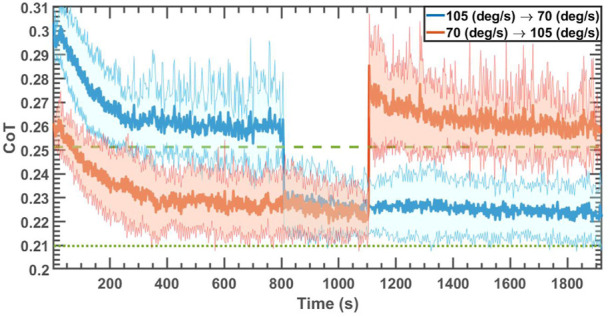
CoT of the robot trotting on uneven ground with the slope of 0 ± 0.5° and ground stiffness of 100 ± 10 kN/m when *ω* changed from 105 deg/s to 70 deg/s and *vice versa* abruptly after 300 blocks. The dashed and dotted lines represent the optimal values for *ω* = 105 deg/s and *ω* = 70 deg/s, respectively.

This set of simulations were designed to investigate the effect of the abrupt changes in the angular velocity of the hip/shoulder joints while the robot was trotting and the ability of the algorithm to converge to near-optimal CoT values after the change. The ground stiffness for these simulations was chosen to be 100 ± 10 kN/m and the longitudinal slope of the ground was 0 ± 0.5°. The angular velocity changed from 105 deg/s to 70 deg/s halfway through each run for one set of simulations, and *vice versa* (70 deg/s to 105 deg/s) for another set. Each simulation contained 600 blocks. Figure 6 displays the average CoT values of 50 independent simulations with solid lines and their standard deviations with the shaded area for each case. The learning rates were set the same as in the simulation setups in section 4.1.3; Table 3 reports the statistics of the last 100 blocks before the abrupt change of the angular velocity (first row of each set) and the final 100 blocks for each case (second row of each set). According to Table 3, the algorithm could converge to the values within 6.67% and 3.5% of the optimal values after abruptly reducing the angular velocity (set 1) and increasing it (set 2), respectively.

**TABLE 3:** Steady-state statistics of Figure 6.

	*ω* (deg/s)	CoT	*r* (deg)	$P_{td}\left(\times 10^{5}\right.$ Pa)
Set 1	105	0.2597 ± 0.0161	14.70 ± 0.49	2.65 ± 0.34
	70	0.2238 ± 0.0088	13.72 ± 0.32	2.56 ± 0.26
Set 2	70	0.2256 ± 0.0099	13.10 ± 0.41	2.53 ± 0.33
	105	0.2602 ± 0.0142	14.55 ± 0.57	2.73 ± 0.37

### 4.2 Experiments on a robot platform

#### 4.2.1 Setup

A number of experiments were conducted using the robot of Figure 1 trotting on a treadmill. These experiments were performed to show the effectiveness of the online learning algorithm on a real platform. The robot used for these experiments is the UPed Gurney et al. (2023), an under-actuated quadruped robot platform explained in Section 3.1. The robot used PID controllers to drive the motors in the hip/shoulder joints, and generate sufficient torque to track desired angular trajectories. The PID controller as well as the online learning algorithm were loaded onto a dSpace MicroLabbox (Paderborn, Germany), a control prototyping system, which provided all motor and pneumatic control to the robot, and set the gait and compliance parameter values.

#### 4.2.2 Performance benchmarks

**FIGURE 7:**
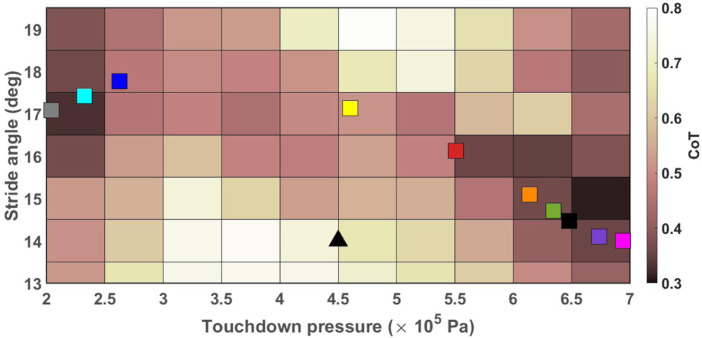
CoT values of the robot trotting on flat ground with *ω* = 105 deg/s as a function of *P*_*td*_ and *r*. The colored squares show the end point of each run. All runs started from the black triangle.

The CoT of the UPed was calculated for the robot trotting with the angular velocity of 105 deg/s and 70 deg/s on a treadmill with longitudinal slopes of 0 and −6° with the stride angle between 12° and 19.5° in steps of 1° and touchdown pressure between 2 × 10^5^ and 7 × 10^5^ Pa in steps of 0.5 × 10^5^ Pa. Figure 7 displays CoT values, as a function of *r* and *P*_*td*_, of the robot trotting on a flat treadmill with *ω* = 105 deg/s. There are two local minima for this case corresponding to CoT values of 0.3090 when (*r*, *P*_*td*_) = (14.5, 7) and 0.3274 when (*r*, *P*_*td*_) = (16.5, 2). Depending on the initial conditions, the algorithm could converge to either of them. Similar measurements for the other 3 cases were performed and the corresponding global and local minima are reported in Table 4.

**TABLE 4:** Experimental setups, and empirically measured values and parameters of the global and local minima.

Simulation parameters		Global minimum			Local minima		
Slope	*ω*	CoT	*r*	*P*_*td*_	CoT	*r*	*P*_*td*_
(deg)	(deg/s)		(deg)	(× 10^5^ Pa)		(deg)	(× 10^5^ Pa)
0	105	0.3090	14.5	7	0.3274	16.5	2
−6	105	0.2850	15	6.5	-	-	-
0	70	0.1717	12	3.5	0.1928	12	6.5
					0.2525	15	5.5
−6	70	0.1215	14	4.5	0.1684	15	6.5

As expected, and similar to the simulation results, Table 4 indicates that the optimal CoT values are different for different forward velocities and longitudinal slopes. Further, similar to biological models, our robot also adapts its stride angle and touchdown pressure to improve its energy efficiency in different ground conditions and operating environments. For example, similar to what has been observed in humans Kim and Park (2011); Elliott and Blanksby (1979), analysis of the experimental results indicate that the optimal values of the stride angle and the stiffness of the leg of the robot are also higher in higher velocities.

#### 4.2.3 Online learning algorithm on the robot platform

Our online learning algorithm was implemented on the UPed in operating conditions tabulated in Table 4. For each experimental setup, 10 independent runs were performed. The initial conditions were chosen to be far from the optimal values. On flat ground, the initial conditions for *ω* = 70 deg/s and *ω* = 105 deg/s were equal to (*r*, *P*_*td*_) = (17.5, 3) and (*r*, *P*_*td*_) = (14, 4.5), respectively. On the downhill slope, the initial conditions for *ω* = 70 deg/s and *ω* = 105 deg/s were equal to (*r*, *P*_*td*_) = (15, 4) and (*r*, *P*_*td*_) = (18, 6.5), respectively. The learning rates for all cases were set to $\mu_{r}=\frac{0.3}{CoT\left(1\right)}$ degrees and $\mu_{P}=\frac{0.15}{CoT\left(1\right)}\times 10^{5}$ Pa. The length of each run was approximately 600 s, but the number of blocks varied from run to run because of the variations in the stride angle due to adaptation and various hip/shoulder angular velocities.

Because the performance surface is multi-modal, the algorithm is only guaranteed to converge to one of the local minima of the surface. Table 5 summarizes the average and standard deviation values of the measured CoT, stride angle and touchdown pressure, computed over the last 60 blocks of each run, and the number of runs *N*_*r*_ that was determined to have converged to each local minimum. For the case of flat terrain and *ω* = 105 deg/s, Figure 7 displays the end points of each of the ten runs (averaged over the last 60 blocks.) Six of the ten runs converged (or is close to convergence) to the global minimum, and three converged to the local minimum of the CoT surface. Analysis of the trends of the parameters of the remaining run suggested that, at the end of the run, the stride angle was reducing and the touchdown pressure was increasing, indicating that the system was traversing toward the global minimum, but may have needed a much longer run time to get there. The cost of transport associated with the global and local minima were relatively close to each other, and the online learning algorithm reduced the CoT from its initial value of 0.59 by more than 45% in all nine cases that converged to one of the two minimum locations. Even the single run that had not converged, resulted in a substantial reduction of the CoT value from 0.58 to 0.43, indicating that our approach is capable of achieving significant improvement in energy efficiency.

**TABLE 5:** Experimental setups and steady-state statistics.

Experimental parameters			Steady-state values		
Slope	*ω*	*N*_*r*_	CoT	*R*	*P*_*td*_
(deg)	(deg/s)			(deg)	(× 10^5^ Pa)
0	105	6	0.3117 ± 0.0187	14.66 ± 0.24	6.41 ± 0.14
		3	0.3534 ± 0.0369	17.44 ± 0.26	2.39 ± 0.32
		1	0.4273 ± 0.0120	17.05 ± 0.16	4.86 ± 0.16
−6	105	10	0.3480 ± 0.0109	16.01 ± 0.21	5.64 ± 0.20
0	70	5	0.2969 ± 0.0097	12.88 ± 0.19	2.66 ± 0.07
		3	0.4143 ± 0.0114	14.43 ± 0.15	5.29 ± 0.15
		2	0.3098 ± 0.0207	13.56 ± 0.19	6.17 ± 0.14
−6	70	3	0.1724 ± 0.0043	16.46 ± 0.19	5.09 ± 0.06
		7	0.1851 ± 0.0051	15.63 ± 0.16	6.21 ± 0.06

**FIGURE 8:**
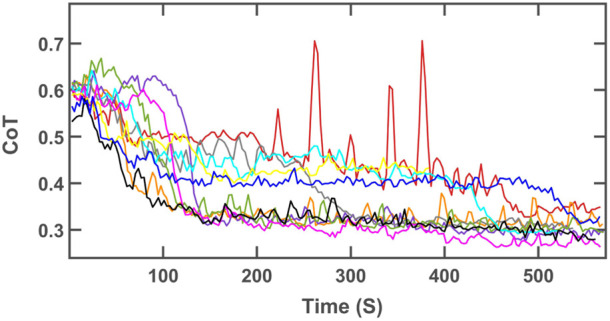
CoT of the robot trotting on a flat treadmill with the angular velocity of 105 deg/s for 10 independent runs.

Figure 8 displays the evolution of the CoT for all individual runs for the experimental setup with *ω* = 105 deg/s and zero slope. Each run, depicted in a specific color, started from the black triangle in Figure 7 and ended at the square with the same color as the trajectory shown in Figure 8. As explained earlier, the run shown in yellow in the two figures had not converged at the end of the run, but still showed a substantial reduction in the cost of transport. The cost of transport of the run shown in red displayed somewhat erratic behavior in the interval between 200 and 400 s Of the run. During this time, the parameters wandered between two possible convergence locations. However, the system could escape this “unstable” region and converge to near-optimal value by the end of the run.

According to Table 4, the CoT values at the local minimum of *ω* = 105 deg/s and zero slope was very close to the global minimum. Consequently, the CoT values are near-optimal values even when the online learning algorithm converges to the local minimum rather than the global minimum. In the angular velocity of 70 deg/s, the algorithm converged to the other local minima in some runs which have higher CoT values compared to the global minima. However, the algorithm could still reduce the energy consumption by as much as 50% compared to the initial conditions.

In most cases, the trends of the optimum values of adaptive parameters observed for both UPed and its simulated model were similar. For example, the optimum values of stride angle, touchdown pressure and CoT increase with increasing forward velocity. However, the CoT values of UPed were greater compared to its model in similar operating conditions and the optimal values of stride angle and the touchdown pressure were different for the actual robot and the simulated robot. One reason for the mismatch between the actual robot and the simulated model of it is that estimating the parameters of the robot precisely is not a trivial task. Even with precise estimation of system parameters, they vary over time. In spite of these differences, we can observe from Tables 2, 4, 5 that both simulation and the actual robot have the same order of magnitude for the CoT values. Therefore, we tuned the hyper-parameters of the simulation model by searching over a wide range of parameters and employed similar values of hyper-parameters for the experiments on UPed.

## 5 Concluding remarks

An online learning algorithm based on gradient descent was presented in this paper to adaptively reduce the energy consumption of a quadruped robot trotting on grounds with unknown characteristics. This algorithm updated the stride angle and leg stiffness in real-time while the robot was trotting. The online learning algorithm was implemented on a simulated model of an under-actuated quadruped robot and also experimentally evaluated using the UPed platform. The algorithm was computationally efficient for implementation in real-time. Different operating conditions associated with different hip/shoulder angular velocities, ground characteristics, and longitudinal slopes were explored. Performance evaluation using simulations and experiments suggested that the online learning algorithm presented in this paper was capable of converging to near-optimal values of the cost of transport for given operating conditions, terrain properties, and gait characteristics.

The approach presented in this paper is a general framework that works without training and can be implemented on any legged robots with adaptive parameters and for any gait, and can also be generalized to optimize more than two parameters. Examples of other such parameters include knee/elbow stiffness, knee/elbow rest angle and asymmetry in the stiffness of the forelegs and the rear legs. Analyses of a model of a horse galloping in a simulation environment (Herr and McMahon, 2001) and the data taken from actual horses (Heglund and Taylor, 1988) have showed that the optimum values of the stride angle and the stride frequency that minimized the CoT vary with forward velocity. Additionally, Wei et al. (2015) demonstrated with a simulated model of a galloping quadruped robot that the leg stiffness should be adjusted for different forward velocities to reduce the CoT. The online learning algorithm presented in this paper can be implemented on fast gaits such as galloping to reduce the CoT of legged robots by updating changeable parameters of their legs. The hyper-parameters of the update equations should be tuned appropriately when applied to other robots and gait conditions. The algorithm can also be modified by using different functions for *f*_*r*_, *f*_*P*_, and different values of *d* in (6) and 7. A simplified model using *d* = 1, and *f*_*r*_ and *f*_*P*_ as signum functions was presented in Aboufazeli (2018) on a model of a quadruped robot similar to the one presented in this paper.

The results of the online learning algorithm showed that adaptively updating the leg stiffness and the stride angle during locomotion reduces the CoT in various ground conditions and forward velocities. Several observations reported in Kim and Park (2011); Farley and Gonzalez (1996); Elliott and Blanksby (1979); Barrey et al. (2002); Jayes and Alexander (1978); Shen and Seipel (2015); Umberger and Martin (2007); Lussiana et al. (2015); García-Pinillos et al. (2019); Telhan et al. (2010) show that animals and humans adjust their leg stiffness and stride angle in various ground conditions and forward velocities. Our results in both simulations and experiments agreed with these observations and demonstrated the usefulness of real-time updates of the stride angle and leg stiffness in legged robots.

## Data Availability

The original contributions presented in the study are included in the article/supplementary material, further inquiries can be directed to the corresponding author.
